# Microstructure and Mechanical Properties of 1080 Plain Carbon Steel Fabricated by Laser Powder Bed Fusion Under High-Density Printing Parameters

**DOI:** 10.3390/ma19061055

**Published:** 2026-03-10

**Authors:** Zechang Zou, Xudong Wu, Cuiyong Tang, Xueyong Chen, Ke Huang

**Affiliations:** 1 School of Mechanical and Intelligent Manufacturing, Fujian Chuanzheng Communications College, Fuzhou 350007, China; 2 College of Mechanical and Electrical Engineering, Fujian Agriculture and Forestry University, Fuzhou 350002, China; 3 College of Materials Science & Engineering, Sichuan University, Chengdu 610065, China

**Keywords:** 1080 plain carbon steel, laser powder bed fusion, microstructure, mechanical properties

## Abstract

For structural metallic materials, performance enhancement has traditionally relied on complex adjustments of chemical composition and heat treatment processes. However, these approaches are complex, costly, and lack sustainability. Metal additive manufacturing (AM) has unique cooling characteristics, providing it with a distinctive approach. In this study, laser powder bed fusion (LPBF) technology was used to prepare high-performance 1080 carbon steel. The study selected three groups of process parameters (VED = 92.59 J/mm^3^) with high density (relative density > 98%) and achieved excellent mechanical properties: the ultimate tensile strength (UTS), yield strength (YS), and elongation (EL) reach 1745.4 MPa, 1455.13 MPa, and 6.77% respectively. The effects of process parameters on microstructure and mechanical properties were investigated. It is found all specimens exhibited a characteristic martensitic needle-like grain morphology without significant crystallographic texture. The microstructure displayed substantial changes as VED varied, with martensite content progressively decreasing with increasing VED. Correspondingly, as the VED increases from 92.59 J/mm^3^ to 225.69 J/mm^3^, the UTS, YS, and EL decrease by 39.0%, 36.1%, and 3.4%, respectively. This work demonstrates the feasibility of achieving high-performance metallic components by precisely controlling additive manufacturing process parameters to manipulate the microstructure of simple alloys, thereby eliminating the need for complex alloying or post-processing heat treatments.

## 1. Introduction

Engineering alloys like steels and aluminum alloys are widely used in applications requiring high strength and damage resistance [1,2]. Engineers and designers frequently improve material performance through intricate processes, such as regulating alloying elements and implementing subsequent heat treatment [3,4,5]. However, recent studies by Tan et al. [6] have demonstrated that a plain carbon steel with an extremely simple composition (Fe and 0.75 wt.% C) can achieve excellent mechanical properties solely through the optimization of additive manufacturing process parameters. This approach is relatively new; therefore, this study aims to further investigate how process parameters affect the microstructure and mechanical properties of plain carbon steel.

Plain carbon steel has long been regarded as an extremely important engineering material [7,8]. During the preparation of steel, various phase transformations occur, forming microstructures with different crystal structures and morphologies, which in turn result in different mechanical properties [9,10,11]. With the surging demand for high-strength materials, high-carbon steel stood out due to its excellent strength, hardness and wear resistance [12]. In the early 20th century, metallurgists made 1080 plain carbon steel an ideal choice for tools, springs, and mechanical parts by precisely controlling the carbon content (0.75–0.88 wt.%) and optimizing the heat treatment process [13]. Due to its simple structure and low cost, 1080 is an important material for agricultural machinery [14,15]. 1080 plain carbon steel is primarily composed of iron and carbon, with carbon playing a dominant role in the eutectoid transformation, thereby critically influencing the material’s hardness and strength [16]. Trace elements are added to assist in achieving different properties, such as Si as a deoxidizer to strengthen the ferrite phase and Mn to counteract the harmful effects of S and improve hardenability [17]. The conventional production of 1080 plain carbon steel involves multiple sequential steps—including smelting, casting, hot rolling, cold working, and heat treatment—to achieve the desired strengthening effect [18].

The limitations of conventional 1080 plain carbon steel production arise from challenges in precise process control and microstructural inhomogeneity, making it difficult to meet the demands of modern industry for compositional accuracy, performance stability, environmental sustainability, and cost-effectiveness. For instance, Alok Mishra et al. [19] conducted cyclic heat treatment on annealed AISI 1080 plain carbon steel bars for different cycles and observed the evolution of their microstructure, achieving higher hardness (845 HV), strength (UTS = 1609 MPa), and equivalent ductility (elongation = 8%). W.D. Kaplan et al. [20] characterized the mechanical damage mechanism of TiC-1080 plain carbon steel cermet using transmission electron microscopy and determined that quasi-static failure occurred due to blunting of interface defects and interface cracking, which then propagated into the steel matrix. Dynamic loading would cause additional failure mechanisms such as transgranular TiC cracking without extending into the steel matrix. You Wang et al. [21] systematically studied the wear mechanism and wear behavior of various microstructures in 1080 plain carbon steel through wear tests and examination of the worn surfaces and wear particles. T.C. Shelton et al. [22] revealed the coarsening mechanism of cementite in 1080 plain carbon steel at different temperature gradients. These studies also indicate that complex heat treatment processes and the addition of other elements and metal compounds are necessary in the preparation of 1080 plain carbon steel to achieve microstructure control and high strength, hardness and wear resistance.

In recent years, additive manufacturing (AM), also known as 3D printing, has developed rapidly [23,24] and has been applied in various fields, such as aerospace, tools, biomedical, and information technology [25,26]. Additive manufacturing (AM) technology primarily involves the geometric design of components, followed by slicing through specialized software, and subsequently building the parts layer by layer along a predefined scan path or pattern to fabricate three-dimensional structures [27,28]. Laser powder bed fusion (LPBF) is one of the most widely used and mature metal additive manufacturing technologies [29]. This process generates extremely high cooling rates, up to 10^4^–10^6^ K/s, during part processing [30]. Therefore, the diffusion of elements is greatly suppressed, which further facilitates the formation of non-equilibrium phases and the refinement of grain size. Wei Chen et al. [31] used additive manufacturing technology to prepare martensitic stainless steel-based composites and achieved an optimized combination of high strength and greater ductility through the addition of micron-sized TiC, with the high strength attributed to grain refinement during the printing process. Raiyan Seed et al. [32] utilized laser powder bed fusion technology to produce ultra-high-strength martensitic steel with a strength as high as 1.4 GPa and an elongation of up to 10.9%. The average equivalent diameter of martensitic laths was only 6.3 ± 1.5 μm. The above research has fully demonstrated the feasibility of LPBF technology in enhancing the strength of steel and refining the grain size. This is highly beneficial for the preparation of 1080 plain carbon steel, enabling the formation of fine-grained and non-equilibrium phase structures such as martensite. At the same time, additive manufacturing also has certain advantages in terms of cost control. When comparing additive manufacturing with traditional processes for producing 1080 steel, although the cost of powder for additive manufacturing is higher (additive manufacturing powder is approximately 50–60 CNY/kg, while traditional powder is about 10–20 CNY/kg), the material utilization rate of the additive manufacturing process is as high as 90–95%, whereas that of traditional processing technology is only 30–50%. Moreover, traditional processes are less advantageous than additive manufacturing in aspects such as mold formulation and personnel monitoring costs.

Meanwhile, the above research indicates that preparing 1080 plain carbon steel using additive manufacturing technology is still in its early stages, with limited studies focusing on high-strength 1080 plain steel via LPBF. Traditional 1080 steel requires complex alloying (such as by adding ceramic particles [20,33]) or multiple heat treatments (cyclic heat treatment [19]) to achieve high performance. The unique process characteristics of LPBF technology enable the achievement of high strength and certain plasticity in 1080 steel, providing a new approach for the production of cost-effective and high-performance structural components. Moreover, by regulating printing parameters [23,34,35], it is expected to precisely control the phase composition, microstructure distribution, and grain size in 1080 plain carbon steel, thereby achieving customized performance of the steel. Therefore, in this study, 1080 carbon structural steel was prepared using laser powder bed fusion (LPBF) technology under various high-density process parameters, enabling a comprehensive investigation of its microstructure and mechanical properties. The aim is to preliminarily establish the process–microstructure–property relationship, providing practical guidance for further research and application of preparing high-performance carbon steel through additive manufacturing technology.

## 2. Materials and Experimental Methods

### 2.1. Raw Materials

**Figure 1:**
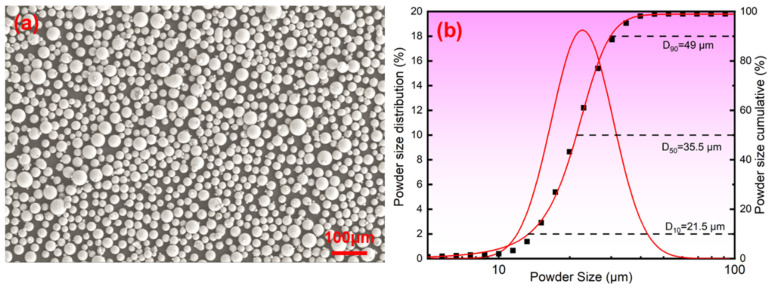
Morphology and size distribution of powder: (**a**) SEM, (**b**) size distribution.

The 1080 spherical powder is provided by Hebei Chuangying Metal Materials Co., Ltd., Shijiazhuang, China, and was prepared by the gas atomization method. The Hall flow rate is 13.2 s/50g. The chemical composition of the 1080 plain carbon steel powder is shown in Table 1. The surface morphology and particle size distribution of the powder are presented in Figure 1a,b respectively. The sphericality of the powder is high (≥98.5%), with a D50 size of 35.50 μm. Its sphericality and appropriate size distribution are very suitable for LPBF technology.

### 2.2. Experimental Methods

**Figure 2:**
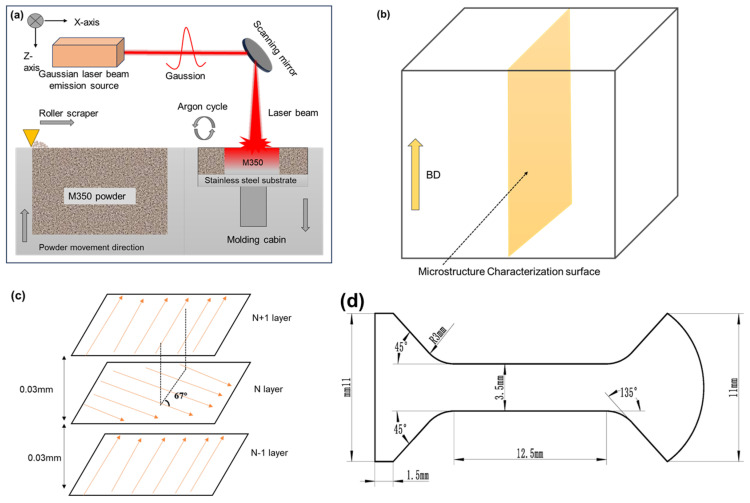
Schematic diagram: (**a**) Principle of LPBF technology, (**b**) scanning strategy, (**c**) microstructure characterization surface, (**d**) tensile specimen size.

The printing process was carried out using the HBD-80 machine produced by Hanbang United 3D Technology Co., Ltd., in Shanghai, China. The equipment parameters were as follows: The machine was equipped with a YLR-500 fiber laser, with a spot diameter of 33 μm and a working wavelength of 1060 nm. Before printing, 1080 powder was placed in a dry box filled with argon gas and left to dry for 8 h to remove any residual moisture in the powder, preventing the formation of air pocket defects during the printing process. During the printing process, argon gas was continuously supplied, and the oxygen content was controlled below 5 ppm to prevent interlayer oxidation during the printing process. The LPBF technology schematic diagram is shown in Figure 2a, the scanning strategy is shown in Figure 2b, and the microstructure characterization surface of the material is shown in Figure 2c. Printing process development was conducted with laser power (P) ranging from 100 to 375 W, scanning speed (V) from 400 to 600 mm/s, hatching distance (H) set at 120 μm, and layer thickness (t) maintained at 30 μm. Simultaneously, the volumetric energy density (VED, defined as VED = P/(H × V × t)) was calculated to reflect the energy input during the printing process to a certain extent, with values ranging from 46.29 to 260.42 J/mm^3^. Among these conditions, three groups of specimens (S1, S2, and S3), which exhibited relatively high density and corresponded to low, medium, and high VED levels, were selected for detailed investigation into the microstructural evolution and mechanical properties of 1080 plain carbon steel under varying process parameters, as shown in Table 2.

### 2.3. Microstructure Characterization

The relative density of this sample was measured using the Archimedes displacement method. The testing instrument used was MAY-D80 (MA-tek Inc., Hayward, CA, USA), with a testing accuracy of 0.001 g/cm^3^. The density condition of the specimen was further observed using a metallographic microscope. The microstructure characterization plane is shown in Figure 2d, parallel to its build direction (BD). The microstructure (texture, phase distribution, and dislocation density, etc.) of the specimen was characterized using the electron backscatter diffraction (EBSD) technique. Before the test, the specimen was polished using the argon ion polishing technique. The electron backscatter diffraction (EBSD) imaging (R-EBSD detector) was performed using the FEI Quanta 200 field emission SEM equipped with EDAX Team (FEI Company, Hillsboro, OR, USA). The EBSD images were retrieved at a voltage of 20 kV and a step size of 250 nm. Finally, the end port morphology of the tensile specimen was photographed and analyzed using the electron scanning microscope (SEM). Perform phase analysis was performed using the Brook D8 DISCOVER X-ray diffractometer (XRD) (Bruker Corporation, Billerica, MA, USA).

### 2.4. Mechanical Properties Test

Three groups of tensile specimens (S1, S2, and S3) were printed under different parameters for tensile testing. The detailed geometric parameters of the specimens are presented in Figure 2d. All tests were conducted at room temperature using a Zwick/Roell universal testing machine with a crosshead speed of 1 mm/min (ZwickRoell, Ulm, Germany). Each set of parameters was tested three times to ensure data consistency. The tensile tests were strictly performed in accordance with the national standard GB/T 228.1-2021 [37].

## 3. Results

### 3.1. Metallurgical Analysis

**Figure 3:**
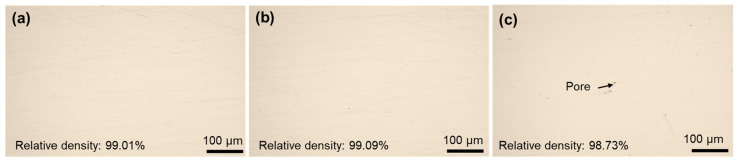
Metallurgical combination situation: (**a**) S1-92.59 J/mm^3^, (**b**) S2-150.46 J/mm^3^, (**c**) S3-225.69 J/mm^3^.

Figure 3 respectively depicts the metallurgical bonding conditions of S1, S2 and S3 specimens with their VEDs 92.59 J/mm^3,^ 150.46 J/mm^3^, and 225.69 J/mm^3^ respectively. From Figure 3, it can be clearly observed that the overall density of the specimens is high, 99.01%, 99.09% and 98.73% respectively. Minor pores were observed in the S3 specimens, likely resulting from keyhole formation induced by the high VED. When studying the regulation of metal microstructure and properties by process parameters, it is necessary to ensure that it is within the dense parameter range. As shown in Figure 3, the high density can largely eliminate the influence of defects on performance. When investigating the regulation of metal microstructure and properties by process parameters, it is essential to operate within the high-density parameter range [38]. As illustrated in Figure 3, a high material density can significantly mitigate the impact of defects on mechanical performance.

### 3.2. Phase Analysis (XRD)

**Figure 4:**
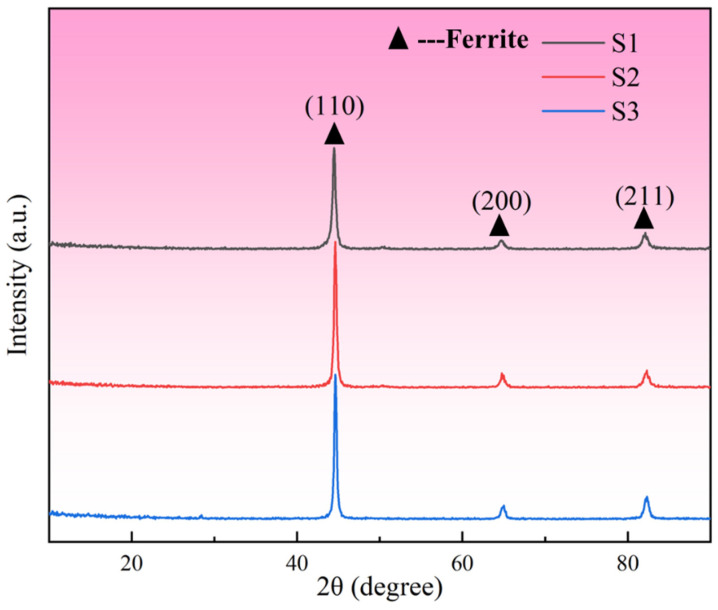
1080 steel phase detection results.

To determine the phase composition of 1080 steel, XRD phase detection was carried out on it, and the test results are shown in Figure 4. The results indicate that the main phase of 1080 steel prepared by LPBF technology is α-Fe, corresponding to the diffraction peaks (110), (200), and (211).

### 3.3. Microstructure

#### 3.3.1. Grain Size Variation

**Figure 5:**
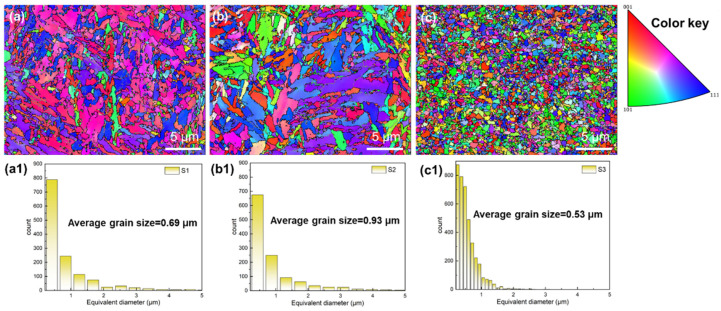
The grain distribution of 1080 plain carbon steel fabricated by LPBF under different process parameters: (**a**,**a1**) S1 (92.59 J/mm^3^), (**b**,**b1**) S2 (150.46 J/mm^3^) and (**c**,**c1**) S3 (225.69 J/mm^3^).

To reveal the microstructure variations of 1080 plain carbon steel under different process parameters, EBSD characterization was conducted on the S1, S2, and S3 specimens. Figure 5 depicts the inverse pole figures (IPF) diagrams and grain distribution histograms of the S1 (VED = 92.59 J/mm^3^), S2 (VED = 150.46 J/mm^3^), and S3 (VED = 225.69 J/mm^3^) specimens. In the figure, different colors represent grains with distinct crystallographic orientations: red corresponds to the <001> orientation, green to <101>, and blue to <111>. As the VED increases, the inverse pole figure (IPF) color distribution undergoes significant changes. For specimen S1, red dominates the color map, indicating <001> as preferred orientation. In contrast, the color distributions of S2 and S3 become progressively more randomized, suggesting that the preferred orientation weakens or disappears with increasing VED. The grain size increases from 0.69 μm to 0.93 μm, and then decreases to 0.53 μm. In a study by P. Lejcek et al. [39]. on pure iron SLM preparation, it was observed that the grain size first increased and then decreased with the energy input, which was consistent with the grain size evolution of 1080 steel in this study (0.69 → 0.93 → 0.53 μm). The grain size growth mechanism will be discussed in discussion Section 4.1.

**Figure 6:**
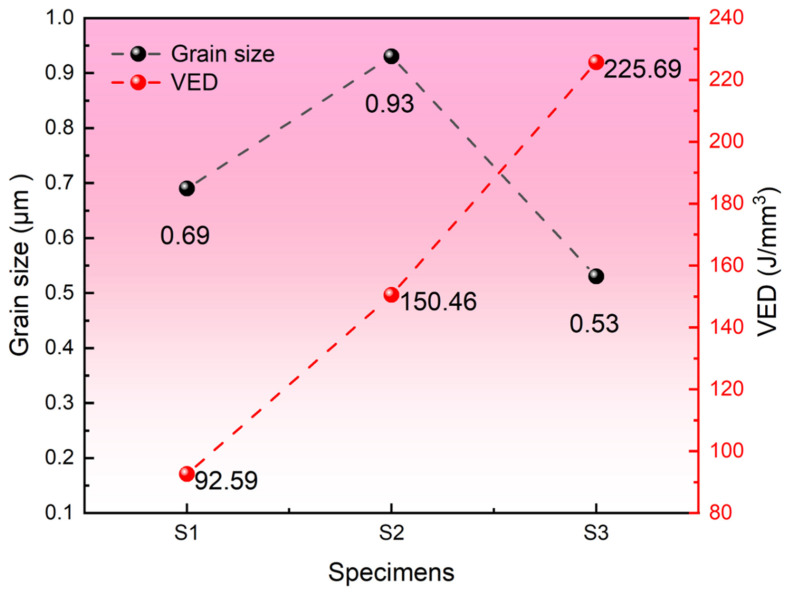
The average grain size variation of 1080 plain carbon steel manufactured by LPBF under different process parameters.

Different processing parameters will have different effects on the microstructure of the material [40,41]. To more intuitively demonstrate the differences in grain size between the S1, S2 and S3 specimens, Figure 6 presents the grain size and volumetric energy density of the three groups of specimens in a dual-*Y*-axis format. It can be clearly observed that the grain size shows a trend of increasing first and then decreasing, and no obvious linear trend is found. A comparison between specimens S1 and S2, both fabricated at a constant scanning speed of 600 mm/s, shows that increasing the laser power from 200 W to 325 W leads to grain coarsening, with the average grain size increasing from 0.69 μm to 0.93 μm. In contrast, at a fixed laser power of 325 W, a decrease in scanning speed from 600 mm/s to 400 mm/s results in significant grain refinement, with the grain size decreasing from 0.93 μm to 0.53 μm, by comparing between S2 and S3.

**Figure 7:**
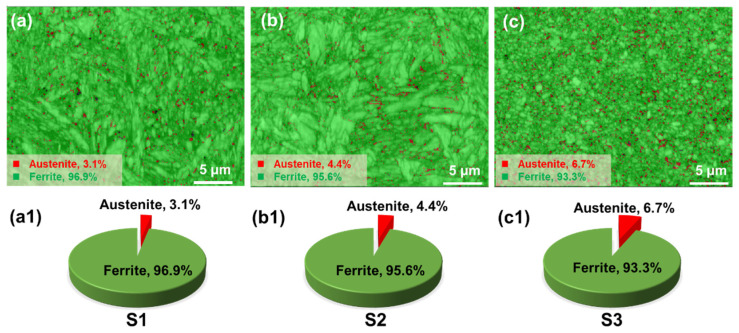
Phase composition and distribution: (**a**,**a1**) S1 (92.59 J/mm^3^), (**b**,**b1**) S2 (150.46 J/mm^3^) and (**c**,**c1**) S3 (225.69 J/mm^3^).

Figure 7 presents the phase compositions of the specimens under different VED conditions. The results show that the ferrite and austenite contents in S1 are 96.9% and 3.1%, respectively; in S2, they are 95.6% and 4.4%, respectively; and in S3, 93.3% and 6.7%, respectively. As the VED increases, the ferrite content in the specimens gradually decreases, while the austenite content gradually increases. Most of the austenite is distributed at the grain boundaries of the ferrite. Because as the VED increases, the energy input gradually increases, the temperature gradient in the molten pool increases, and the coupling effect of heat dissipation speed leads to a greater diffusion driving force for carbon in the steel. As is well known, the formation of martensite is a non-equilibrium supersaturated solid solution of carbon in ferrite. The faster the cooling speed, the more conducive it is to the formation of martensite, and the increase in VED actually reduces the cooling speed, which leads to a decrease in the formation of martensite and an increase in the residual austenite content. This viewpoint has also been mentioned in reference [42].

#### 3.3.2. Crystallographic Texture Variation

**Figure 8:**
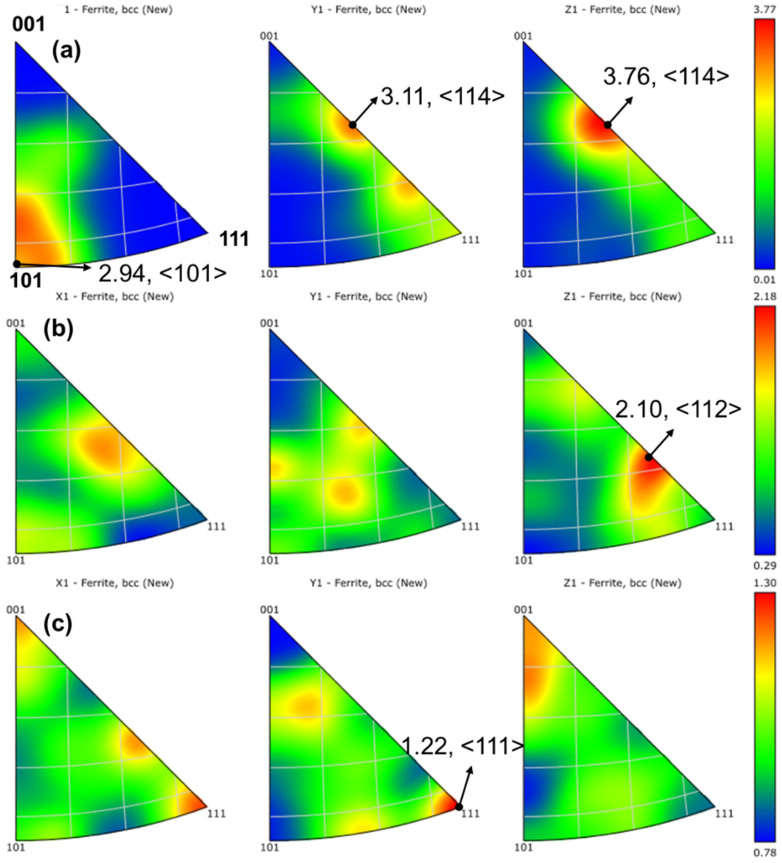
Inverse pole figures (IPFs) of LPBF-fabricated 1080 plain carbon steel at different process parameters: (**a**) S1 (VED = 92.59 J/mm^3^), (**b**) S2 (150.46 J/mm^3^), (**c**) S3 (225.69 J/mm^3^).

Figure 8 depicts the inverse pole figures of specimens S1, S2, and S3, which can to some extent reflect the preferred orientation relationship of the crystals in the specimens. As shown in Figure 8a, a strong <114>//Y, <114>//Z texture and a weak <101>//X texture were observed. When VED = 150.46 J/mm^3^, a strong <112>//Z texture appeared in Figure 8b, while no obvious strong orientation was observed in the other two directions. As VED further increased, the crystal orientation changed again, becoming a strong <111>//Y texture, which was also reflected to some extent in the IPF diagram of Figure 5. Clearly, the textures of 1080 plain carbon steel manufactured under different parameters show significant variations. The increase in VED contributes to the reduction in crystallographic texture and enhances microstructural homogeneity.

#### 3.3.3. Kernel Average Misorientation Variation

**Figure 9:**
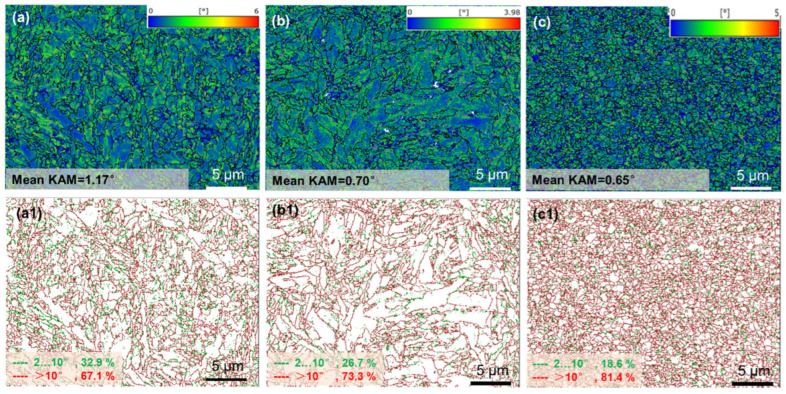
KAM figures (**a**–**c**) and distribution of grain boundary angle (**a1**–**c1**) of LPBF-fabricated specimens at different process parameters: (**a**,**a1**) S1 (VED = 92.59 J/mm^3^), (**b**,**b1**) S2 (150.46 J/mm^3^), (**c**,**c1**) S3 (225.69 J/mm^3^).

In the LPBF process, due to its unique thermal history and rapid solidification rate, obvious thermal stress accumulation is inevitable during the printing process, leading to plastic deformation within its microstructure [29]. The average orientation difference kernel average misorientation (KAM) value can quantify this degree, indicating the level of thermal stress concentration or defect density [38]. Figure 9 depicts the KAM diagrams and grain boundary angle distribution diagrams of specimens S1, S2, and S3. In Figure 9a1–c1, the green lines represent low-angle grain boundaries (2–10°), and the red ones represent high-angle grain boundaries (>10°). It can be observed that as VED changes from 92.59 J/mm^3^ to 225.69 J/mm^3^, the proportion of low-angle grain boundaries gradually decreases to approximately 14.3%. The low-angle and high-angle grain boundaries decrease from 32.9% (S1-92.59 J/mm^3^) to 18.6% (S3-225.69 J/mm^3^) and increase from 67.1% (S1-92.59 J/mm^3^) to 81.4% (S3-225.69 J/mm^3^). From the perspective of KAM values, as VED increases, the KAM of the specimens shows a gradually decreasing trend (S1-1.17°, S2-0.70°, and S3-0.65°), mainly attributed to the LPBF process, whereas VED increases, the cooling rate of the molten pool gradually decreases, and the temperature gradient increases, which is similar to the tempering process in heat treatment, effectively reducing stress accumulation and other defects in the crystals. KAM decreased sharply from 92.59 to 150.46 J/mm^3^: at low VED (S1), the cooling rate was high, and thermal stress accumulated rapidly and was difficult to release, resulting in a high KAM value (1.17°); at medium VED (S2), the cooling rate decreased, and interlayer reheating (in situ tempering) alleviated some of the thermal stress, causing KAM to drop sharply to 0.70°. KAM decreased gradually from 150.46 to 225.69 J/mm^3^: at high VED (S3), the cooling rate further decreased, but the phase transformation stress generated during the γ → α phase transformation partially counteracted the release of thermal stress, resulting in only a slight decrease in KAM to 0.65°. Huang et al. [29] also reported a similar phenomenon, that is, within a certain range, there is a linear correlation between KAM and VED.

**Figure 10:**
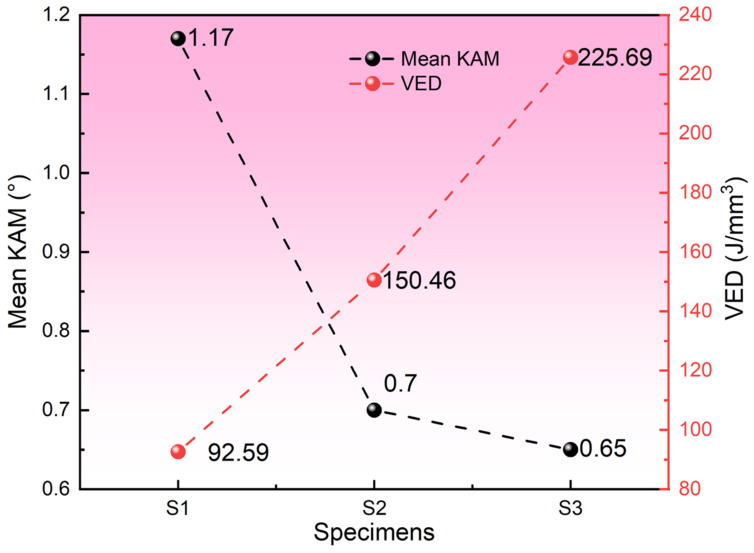
Relationship between mean KAM and VED.

Figure 10 presents the KAM values and corresponding VED levels for specimens S1, S2, and S3, quantitatively characterizing the variations in micro-stress accumulation within 1080 plain carbon steel under different processing conditions. As previously discussed, the KAM values exhibit a significant decrease with increasing VED, and the underlying mechanisms for this trend have been analyzed in detail in earlier sections.

### 3.4. Mechanical Properties

**Figure 11:**
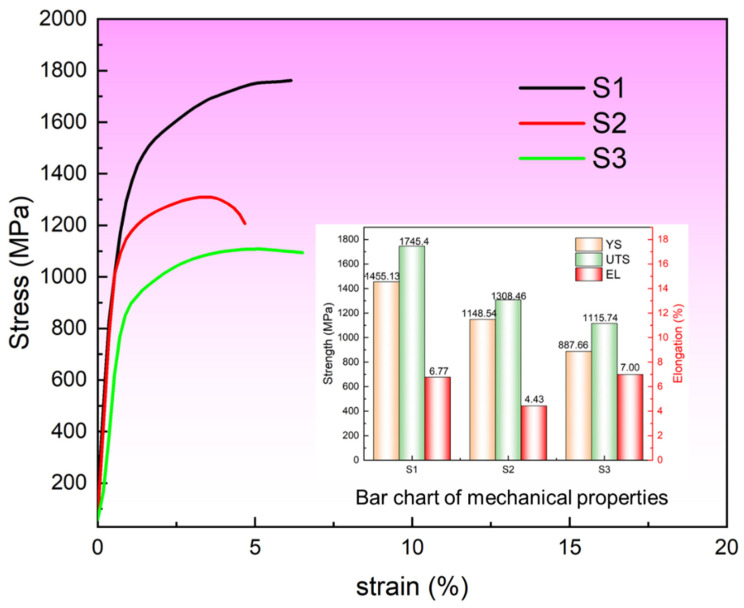
Stress–strain curves of S1, S2 and S3 specimens.

The strength and ductility are critical indicators for evaluating the mechanical performance of materials. For each processing condition, three repetitions of tensile tests were conducted, and three representative stress–strain curves are plotted in Figure 11. The inset figure in Figure 10 shows the measured strength, elongation, and standard deviation of the three groups of specimens. The small standard deviations indicate good repeatability and reliability of the experimental data. As shown in Figure 11, the yield strength (YS) of specimens S1, S2, and S3 remains at a relatively high level but exhibits a decreasing trend, with values of 1455.13 MPa, 1148.54 MPa, and 887.66 MPa, respectively. However, in terms of elongation, the specimens exhibit a trend of initially decreasing followed by an increase, with elongation values of 6.77%, 4.43%, and 7.00% for S1, S2, and S3, respectively. This follows a trend similar to that of the grain size. The refinement of grain size enhances plastic deformation through cooperative grain boundary sliding and intragranular deformation, thereby improving the ductility of the specimen. The observed differences in mechanical properties primarily stem from variations in grain size, dislocation density, and phase composition under different VED conditions. Notably, the relative contributions of these factors to the overall material strength differ. A detailed quantitative analysis of these strengthening mechanisms is provided in Section 4.

## 4. Discussion

### 4.1. Microstructure Evolution

In this study, three sets of process parameters (S1 = 92.59 J/mm^3^, S2 = 150.46 J/mm^3^, and S3 = 225.69 J/mm^3^) were investigated in detail. Figure 5 clearly shows that as VED changes from 92.59 J/mm^3^ to 225.69 J/mm^3^, the grain size changes from 0.69 μm to 0.93 then to 0.53 μm. The phenomenon of grain size increasing with the increase in VED can be explained by the classical solidification theory [43]. As is well known, the morphology of grains is mainly influenced by the ratio of temperature gradient (G) to solidification rate (R) during the solidification process. The larger the ratio of G/R, the more conducive it is to the formation of columnar crystals and the formation of larger-sized grains [42]. This aspect is mainly determined by the input energy of the laser in the LPBF process. In this study, the energy input of specimens S1, S2, and S3 gradually increased, which led to a decrease in the cooling rate (R) and an increase in the temperature gradient (G) during the solidification process. This resulted in an increase in the ratio of G/R, thereby causing further growth of the grains.

On the other hand, the phenomenon of equiaxed fine grains observed in the S3 specimen may result from phase transformation. It is well known that during the solidification process of 1080 steel, there is a transformation process from the δ to γ phase, and from the γ to α phase [44]. In the LPBF process, due to the fast cooling speed of the process itself, the α phase grains grow directly from the molten pool without going through the previous phase transformation steps. However, when the laser beam scans over the previous layer again, it inevitably re-heats to the molten state. This causes the original columnar grains to transform into γ and some δ phase, and then cool back to α phase. At this time, due to the change in the lattice parameters of the individual structure, these changes are closely related to the volume changes, which in turn leads to an increase in internal strain [45]. This change can be reduced through the self-catalytic nucleation in front of the γ/α interface of the migrating ferrite grains during the cooling process. As a result, the phase structure in the solidification molten pool undergoes a complex transformation, causing the columnar grains to decompose into smaller and equiaxed grains [46]. In the S3 specimens, the high VED further promotes this effect, leading to the formation of equiaxed fine grains. This phenomenon is similar to that observed by Zhang [47]. In summary, the unexpected increase then decrease in grain size with increasing VED is attributed to the competing effects of solidification dynamics and phase transformation mechanisms.

Figure 6 and Figure 8 illustrate the variations in phase composition and KAM values of the specimens. The results show that the microstructure is predominantly composed of ferrite, with a small fraction of retained austenite. As the VED increases, the austenite content gradually rises, a phenomenon that is characteristic of the LPBF process. This behavior is analogous to the tempering process in conventional heat treatment. During LPBF, molten metal powder is deposited layer by layer. With each subsequent layer, the underlying layers are inevitably reheated due to the thermal input from the newly melted material, effectively subjecting the previously solidified layers to a form of in situ heat treatment. In this study, as the VED increased across specimens S1, S2, and S3, the overall heat input also increased, resulting in a progressively stronger thermal influence on the underlying layers. It is well known that martensite forms as a supersaturated solid solution derived from austenite during rapid cooling. The high cooling rates inherent to the LPBF process promote significant martensite formation. However, increasing VED elevates the interlayer temperature, enhancing carbon diffusion, which in turn reduces martensite content and promotes the retention of austenite during solidification.

### 4.2. Reason for Differences in Mechanical Properties and Strengthening Mechanisms

According to the Hall-Petch strengthening effect [23,42], grain refinement leads to an increase in strength, but the strength is not limited to the contribution of grain size. In 1080 plain carbon steel, there are two phases (ferrite and austenite), and as the VED increases, the austenite content increases, which inevitably leads to a decrease in strength. Meanwhile, it can be observed in Figure 9 that as VED increases, the KAM value decreases, which leads to the weakening of the second-phase strengthening effect and the dislocation strengthening effect in the S3 specimen, and the coupling of the two ultimately leads to a significant decrease in strength, which has also been mentioned in the literature [6]. Beyond that, the density variation among the specimens mentioned in Figure 3 could also induced strength debit. This is also visualized in conjunction with the tensile test data in Figure 11. In order to further analyze the contribution of different strengthening mechanisms to the three groups of specimens, the following theoretical contributions will be calculated based on the microstructural data. Material strengthening mechanisms are categorized into four main types (grain boundary strengthening ($\sigma_{GB}$), dislocation strengthening ($\sigma_{GND}$), solid solution strengthening ($\sigma_{s}$) and second-phase strengthening ($\sigma_{sp}$)) of strengthening mechanisms. In the 1080 plain carbon steel manufactured by the LPBF, the contribution of high-density dislocations and grain boundary strengthening to strength is mainly considered. Of course, in the calculation of yield strength contribution, there is solute strengthening caused by trace elements. However, compared with the contribution mechanisms such as grain boundary strengthening and second-phase strengthening in 1080 steel, these contributions are relatively small. They are difficult to be quantitatively characterized, and their effects have been included in the errors of experimental and theoretical calculations.

The yield strength [48] is calculated as presented in Equation (1):

(1) $$\sigma_{y}=\sigma_{0}+\sigma_{GB}+\sigma_{GND}$$

The grain boundary strengthening contribution [49] can be expressed by Equation (2):

(2) $$\sigma_{GB}=\sigma_{0}+kd^{-0.5}$$

where $\sigma_{0}$ and k are Hall-Petch parameters. $\sigma_{0}$ is the lattice friction, which is 50 MPa [48]. k (Hall-Petch constant) is 0.25 MPa·m^−1/2^. d is the average grain size of the grains. According to the EBSD test data, the average grain sizes of S1, S2 and S3 specimens were 0.69, 0.93 and 0.53 μm, respectively. Therefore, their grain boundary strengthening contributions were calculated to be 300.96 MPa, 259.23 MPa and 343.4 MPa, respectively.

The dislocation strengthening contribution can be expressed by Equation (3) [50]:

(3) $$\sigma_{GND}=M\alpha Gb\sqrt{\rho }$$

Here α = 0.3, the fundamental shear modulus is expressed as G = 71 GPa [51] and the Burgers vector is b = 0.25 nm. ρ is the dislocation strength. m = 3 is the Taylor factor. According to the strain gradient theory [52], the KAM value can quantify the dislocation density within the material to a certain extent, and its dislocation density can be calculated theoretically according to Equation (4) [38].

(4) $$GND=\frac{\;2KAM}{\left({Burger}^{\prime }s\;vector\;\cdot step\right)}$$

where the KAM computational equation is converted to the radian system. According to the EBSD test data, the step was 0.0473 μm and b (Burger’s vector) was 0.286 nm. The KAM for the S1, S2, and S3 specimens have been illustrated in Figure 9, which were 1.17°, 0.7°, and 0.65°, respectively. The GND of the three specimens were calculated to be 30.19 × 10^14^/m^2^, 18.06 × 10^14^/m^2^ and 16.77 × 10^14^/m^2^, respectively, which were brought into Equation (3) to calculate the dislocation densities, which were 877.75 MPa, 678.89 MPa and 654.20 MPa, respectively.

Table 3 summarizes the experimentally measured and theoretically calculated yield strengths for the three specimen groups, along with the corresponding error values of 15.56%, 13.96%, and 17.99%, respectively. All errors fall below the 20% threshold, indicating good agreement between experimental and theoretical results with a high level of confidence.

**Figure 12:**
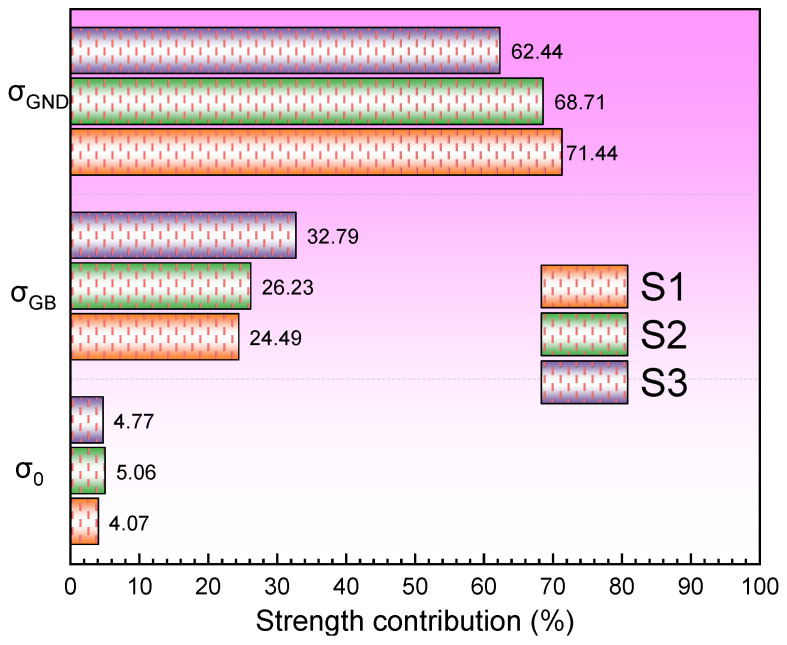
Different types of strength contributions.

Figure 12 further visualizes the percentage contributions of different strengthening mechanisms to the yield strength. The results indicate that the strength differences among the three specimen groups primarily arise from fine-grain strengthening and dislocation strengthening, with dislocation strengthening being dominant in S1, while fine-grain strengthening is most pronounced in S3. During the LPBF process, lower energy input in S1 leads to higher cooling rates, which promote the accumulation of residual stresses and a high density of dislocations. As previously described, S3 exhibits enhanced fine-grain strengthening due to its smaller grain size. Simultaneously, the contribution of dislocation strengthening to the yield strength in S1 exceeds the contribution of fine-grain strengthening in S3. Consequently, the yield strength of S1 is higher than that of S3.

## 5. Conclusions

In this study, high-strength 1080 plain carbon steel was additively manufactured via laser powder bed fusion (LPBF). The variations in microstructure and mechanical properties under high-density processing parameters were systematically examined. The key findings are summarized as follows:

- **Microstructural variations induced by process parameters:** Specimens S1, S2, and S3 were fabricated under VEDs of 92.59 J/mm^3^, 150.46 J/mm^3^, and 225.69 J/mm^3^, respectively. With increasing VED, the grain size exhibited a non-monotonic trend—increasing from 0.69 μm to 0.93 μm and then decreasing to 0.53 μm—reflecting the competing effects of solidification and phase transformation. As VED increases, the density of the sample first increases and then decreases. Excessively large VED will directly lead to the occurrence of circular holes. Moreover, pronounced changes were observed in crystallographic texture, kernel average misorientation (KAM), and phase composition. Specifically, as the VED increased from 92.59 J/mm^3^ to 225.69 J/mm^3^, the KAM value decreased from 1.17° to 0.65°, and the ferrite phase fraction declined from 96.9% to 93.3%.
- **Mechanical property variations induced by process parameters:** The yield strength (YS), ultimate tensile strength (UTS), and elongation (EL) demonstrated pronounced variations across the three VED conditions. Specifically, YS ranged from 887.66 MPa to 1455.13 MPa (ΔYS = 567.47 MPa), UTS from 1115.74 MPa to 1745.45 MPa (ΔUTS = 629.71 MPa), and EL from 4.43% to 7.00% (ΔEL = 2.51%). These results highlight the strong sensitivity of mechanical properties to process parameter optimization in LPBF-fabricated 1080 plain carbon steel.
- **Process–microstructure–properties linkage:** Variations in energy input significantly influenced the grain size, dislocation density, and martensitic phase content of 1080 plain carbon steel. Lower energy input (S1) promoted martensite formation and higher dislocation densities. For S3 specimens with high energy inputs, grain refinement led to an enhanced grain boundary strengthening effect. Among the three specimen groups, dislocation strengthening was the dominant strengthening mechanism. Therefore, S1 owns the highest strength.

Compared with traditional approaches involving complex alloying and heat treatments, high-performance metallic components can also be effectively fabricated by directly optimizing additive manufacturing (AM) process parameters. This study demonstrates the feasibility of tailoring high-performance metal parts through precise regulation of AM processing conditions.

## Figures and Tables

**Table 1:** Chemical composition of 1080 plain carbon steel [36].

Elements	C	Si	Mn	S	P	Fe
wt.%	0.75	0.19	0.79	0.01	0.01	Bal.

**Table 2:** Process parameters of S1, S2, S3 specimens.

No.	P (W)	V (mm/s)	H (μm)	VED (J/mm^3^)
S1	200	600	120	92.59
S2	325	600	120	150.46
S3	325	400	120	225.69

**Table 3:** Yield strength: Theoretical value vs. test value.

Strengthening Type	Strength Contribution (MPa)		
	S1	S2	S3
$\sigma_{0}$	50	50	50
$\sigma_{GB}$	300.96	259.23	343.4
$\sigma_{GND}$	877.75	678.89	654.20
$\mathrm{Theoretical}\mathrm{calculation}\;\sigma_{y}$	1228.71	988.12	1047.4
$\mathrm{Test}\mathrm{value}\;\sigma_{y}$	1455.13 ± 28.17	1148.54 ± 22.32	887.66 ± 36.35
Error	15.56%	13.96%	17.99%

## Data Availability

The original contributions presented in this study are included in the article. Further inquiries can be directed to the corresponding author.
